# Comparative Study of Two Spectral Methods for Estimating the Excited State Dipole Moment of Non-Fluorescent Molecules

**DOI:** 10.3390/molecules29143358

**Published:** 2024-07-17

**Authors:** Mihaela Iuliana Avadanei, Dana Ortansa Dorohoi

**Affiliations:** 1 “Petru Poni” Institute of Macromolecular Chemistry, 41A Gr. Ghica Voda Alley, 700487 Iasi, Romania; mavadanei@icmpp.ro; 2 Faculty of Physics, “Alexandru Ioan Cuza” University, 11 Carol Blvd., 700506 Iasi, Romania

**Keywords:** cycloimmonium ylids, charge transfer band, excited state dipole moment, solvatochromism, intermolecular interactions

## Abstract

The electronic absorption spectral characteristics of cycloimmonium ylids with a zwitterionic structure have been analyzed in forty-three solvents with different hydrogen bonding abilities. The two ylids lack fluorescence emission but are very dynamic in electronic absorption spectra. Using the maximum of the ICT band, the goal was to establish an accurate relationship between the shift of the ICT visible band and the solvent parameters and to estimate two of the descriptors of the first (the) excited state: the dipole moment and the polarizability. Two procedures were involved: the variational method and the relationships of the Abe model. The results indicate that the excited state dipole moment of the two methylids decreases in the absorption process in comparison with the ground state. The introduction of a correction term in the Abe model that neglects the intermolecular H-bonding interactions leads to a more accurate determination of the two descriptors. The strong solvatochromic response of both ylids has been further applied in distinguishing the solvents as a function of their specific parameters. Principal component analysis was applied to five selected properties, including the maximum of the charge transfer band. The results were further applied to discriminate several binary solvent mixtures.

## 1. Introduction

The aim of this study is to analyze the results obtained when estimating the excited state dipole moment of some zwitterionic solute molecules that are spectrally active only in electronic absorption using two known methods: the variational method [1] applied to the solvatochromic results and the theoretical model developed by Takehiro Abe [2] to describe the solvent influence on the wavenumbers in the maxima of the electronic absorption bands of neutral organic molecules. This model was applied by the author’s team to estimate the dipole moments and polarizabilities in the excited states of naphthalene [3].

In 1981, T. Abe developed a new model for the solvent’s effects on the frequency shifts of electronic spectra of anions [4], considering systems containing large anion counters by cations and numerous solvent molecules, such as Janovsky complexes of the potassium salts of 1-acetonyl-1,3-dinitro-2,5-cyclohexadienide, and 4-acetonylidene-1,3-dinitro-2,5-cyclohexadienide.

The theories describing the solvent’s influence on the electronic (absorption and fluorescence) spectra [2,5] establish a series of correlations between the solute descriptors (in their electronic states responsible for the electronic band appearance) and the solvent parameters. These correlations were used when estimating the excited state dipole moment only for those molecules that were spectrally active both in electronic and fluorescence spectra [5,6,7,8,9,10]. The obtained values were, however, affected by the simplifying hypothesis in which the theories were developed. For example, in all theories regarding the solvent influence on the electronic spectra, the specific interactions between the molecules were neglected due to their complexity and local action. This type of interaction is taken into consideration only in empirical terms when added to the theoretically established ones.

Additionally, the other simplifying hypotheses used in each theory determine errors when estimating the excited descriptors of the solute molecules. The different expressions for the terms describing the long-range interactions in the simple liquids, such in [11,12,13,14], compared with [15,16,17,18], cause great differences between the estimated values of the excited states parameters [19,20,21,22], even for the cases of solutes showing both absorption and fluorescence electronic spectra.

For the molecules active only in the absorption spectra, a simple method [1] was developed based on solvatochromic determinations. In this method, the wavenumbers in the maximum of the solute absorption band were measured in a large number of solvents, and a multilinear correlation with the solvent parameters was established. The dependence (established by the theory of solutions) between the correlation coefficients of the linear dependence and the molecular descriptors of the solute were then used to estimate the excited state dipole moment. This method is based on McRae’s hypothesis, which states that the excited polarizability of the solute does not change its value in the absorption process [5,6]. The dependence of the excited state polarizability on the molecular dipole moments is established based on the solvatochromic study. The angle between the dipole moments of the solute in the electronic states of transition is varied until the excited state polarizability equalizes the ground state polarizability. This value of the angle is considered when corresponding to the absorption process and determines the value of the excited state dipole moment.

Because the variational method is based on a restrictive hypothesis, namely, the solute excited state polarizability is equal to the ground state one, we intended to verify the validity of this method for some molecules that are inactive in fluorescence and show absorption bands only, using an alternate procedure based on the Abe model.

The Abe model [2] establishes a series of correlations between the spectral characteristics of the solute and the solvents, based on which one can estimate, using statistical methods, the value of the excited state dipole moment. This theoretical model expresses the spectral shifts from the electronic spectra as being due only to universal interactions (neglecting the specific interactions) and also neglects the angle between the dipole moments of the solute in the electronic states responsible for the absorption process.

Although the two methods are developed for different simplifying hypotheses, we compare the results obtained for estimating the excited state dipole moment of some zwitterionic solutes when their descriptors in the ground electronic states are known.

For this purpose, two zwitterionic molecules from the pyridinium ylid class were chosen. The separated opposite charges of the two ylids were located on the heterocycle and on the carbanion, respectively [23,24]. The carbanion, carrying the negatively charged atom, can be mono- or disubstituted with electronegative atomic groups for increasing molecular stability. These molecular structures have a pronounced basic character and are able to participate in both universal and specific interactions with different solvents [24,25]. In hydroxy solvents, such as acids or alcohols, the pyridinium methylids participate in proton transfer processes between the -OH group of the solvent and their carbanion substituents. They are active only in the electronic absorption spectra and show a visible absorption band that is very sensitive to the solvent’s influence. Attributed to an intramolecular charge transfer (ICT) from the carbanion towards the heterocycle [24,25,26,27,28], this absorption band exhibits a great bathochromic shift when passing from polar to non-polar solvents and even disappears in acid solutions. This ICT process is, therefore, accountable for the lack of fluorescence emission.

A strong relationship between the solvent nature and the electron density has been observed to direct the reactions of pyridinium ylids with various reagents towards specific paths [29,30,31]. Pyridinium ylids are well-known carbon nucleophiles, and in aprotic solvents, they prefer to undergo 1,3-dipolar cycloadditions to afford N-heterocycles [29,30,31,32]. In protic solvents (ethanol or N,N-dimethylformamide), pyridinium ylids may either suffer an elimination reaction or may follow a double path, using 1,3-dipolar cycloaddition simultaneously with an addition–elimination reaction [30]. The protonation of zwitterion in alcohols is sometimes the first stage in the ring closure, leading to dihydrofuran [32] or can be responsible for inhibiting some reaction paths in other cases [30]. The nature of substituents at either the aromatic ring or carbanion influences the course of cycloaddition reactions or [3+2] cycloadditions [33,34,35,36,37]. So, analysis of the electronic properties of a solute, especially in strongly interacting solvents, must be performed with a minimum number of errors. Therefore, this study is very informative about the validity of the methods used in estimating the excited dipole moment of the solute in close relation to the parameters of the solvent.

## 2. Results and Discussions

### 2.1. Theoretical Bases

Spectral studies regarding the solvent’s influence on the electronic bands are conducted with diluted solutions (10^−3^–10^−5^ mol/L) of the spectrally active molecule (solutes) in solvents inactive in the searched spectral range. In these conditions, only the solute—solvent and solvent—solvent interactions influence the position of the electronic bands in the wavenumber scale. The distance between the solute molecules is long and their interactions are neglected in the theoretical description of the interaction energies. The existent theories developed for diluted solutions describe only the universal interactions between the solvent (considered as an infinite, homogeneous and polarizable dielectrics) and solute molecules [2,5,6]. The contribution of the universal interactions on the electronic band positions can be described by functions depending on the solvent electric permittivity, *ε*, and refractive index, *n*, of the type [15,16,17,18]; $f\left(\varepsilon \right)=\frac{\varepsilon -1}{\varepsilon +2}$ and $f\left(n\right)=\frac{n^{2}-1}{n^{2}+2}$.

When the universal (induction, polarization, dispersion) interactions are prevalent in solutions, the relations obtained in the existent theories can be transformed as multilinear functions of the type:

(1) $$\bar{v}\left({cm}^{-1}\right)=v_{0}{\left({cm}^{-1}\right)+C}_{1}f\left(Ɛ\right)+C_{2}f\left(n\right)$$

In Equation (1), the free term indicates the wavenumbers in the isolated state of the spectrally active molecule. The next two terms (theoretically established) describe the contribution of the universal interactions between the solvent (considered as a continuous dielectric) and the solute molecule to the spectral shift of the electronic band.

The wavenumbers computed with relation (1) in the maximum of the electronic bands have different values from the experimental ones. In order to avoid this non-concordance, scientists introduced some empirical parameters [38,39,40,41] to describe the possible specific interactions from liquid solutions. The solvent parameters *α* (acidity parameter) and *β* (basicity parameter) are used to take into consideration specific interactions by hydrogen bonds when the solvent donates or receives protons are defined in [39,40]. The corresponding terms were added in relation (1).

(2) $$\bar{v}\left({cm}^{-1}\right)=v_{0}{\left({cm}^{-1}\right)+C}_{1}f\left(Ɛ\right)+C_{2}f\left(n\right){+C}_{3}\beta {+C}_{4}\alpha$$

The specific interaction influence on the electronic band position is given in Equation (2) by the last terms when the solvent receives or donates a proton, respectively. The correlation coefficients from Equation (2) can be estimated by statistical means [15,16,17,18,25,26,27,28] using known solvent parameters and the wavenumbers obtained in the solvatochromic analysis.

The importance of this model in describing the solvatochromic behavior of the electronic spectra lies in the fact that the correlations coefficients *C*_1_ and *C*_2_ depend on the solute descriptors, as is shown in Equations (3) and (4) [25,26,27,28].

(3) $$C_{1}=\frac{2{µ}_{g}({µ}_{g}-{µ}_{e}cos\varphi )}{a^{3}}+3kT\frac{\alpha_{g}-\alpha_{e}}{a^{3}}$$

(4) $$C_{2}=\frac{{µ}_{g}^{2}-{µ}_{e}^{2}}{a^{3}}-\frac{2{µ}_{g}({µ}_{g}-{µ}_{e}cos\varphi )}{a^{3}}-3kT\frac{\alpha_{g}-\alpha_{e}}{a^{3}}+\frac{3}{2}\frac{\alpha_{g}-\alpha_{e}}{a^{3}}\frac{I_{u}I_{v}}{I_{u}{+I}_{v}}.$$

The notations used in these equations indicate the dipole moment, μ, the electric isotropic polarizability, *α*, the ionization potential, *I*, the temperature *T*, the angle *φ* between the molecular dipole moments in the two electronic states responsible for the visible band appearance, and the solute molecular radius, *a*.

The indices *u* and *v* refer to the solute and solvent molecules, and the indices *g* and *e* refer to the ground and excited state of the solute, respectively. The correlation coefficients $C_{1}$ and $C_{2}$ from Equations (3) to (4) are expressed as erg = 10^−7^ Joule, the dipole moments in ues·cm, the term 3*kT* in erg (*k* = 1.38·10^−16^ erg·K^−1^), and absolute temperature in K.

Very simple mathematical operations provide the following:

(5) $${(C}_{1}+C_{2})a^{3}={µ}_{g}^{2}-{µ}_{e}^{2}+\frac{3}{2}\frac{I_{u}I_{v}}{I_{u}+I_{v}}(\alpha_{g}-\alpha_{e})$$

(6) $$\alpha_{g}-\alpha_{e}=\frac{2}{3}\frac{I_{u}{+I}_{v}}{I_{u}I_{v}}\left[\left(C_{1}+C_{2}\right)a^{3}-{µ}_{g}^{2}+{µ}_{e}^{2}\right]$$

Using the difference $\alpha_{g}-\alpha_{e}$ from (6), one can obtain Equation (7) from Equation (3):

(7) $$2kT\frac{I_{u}{+I}_{v}}{I_{u}I_{v}}{µ}_{e}^{2}-2{µ}_{g}{µ}_{e}cos\varphi +2{µ}_{g}^{2}-C_{1}a^{3}+2KT\frac{I_{u}{+I}_{v}}{I_{u}I_{v}}\left[\left(C_{1}+C_{2}\right)a^{3}-{µ}_{g}^{2}\right]=0$$

In order to give real solutions for the excited dipole moment, μ*_e_*, of the solute molecule, Equation (7) must have a discriminator, Δ, greater than zero.

(8) $$∆={\left(2{µ}_{g}cos\varphi \right)}^{2}-8kT\frac{I_{u}{+I}_{v}}{I_{u}I_{v}}\left\{2{µ}_{g}^{2}-C_{1}a^{3}+2kT\frac{I_{u}{+I}_{v}}{I_{u}I_{v}}\left[\left(C_{1}+C_{2}\right)a^{3}-{µ}_{g}^{2}\right]\right\}$$

The solutions ${{µ}_{e}}_{1,2}$ of Equation (7) depend on the angle *φ*, as it results from (8) and (9).

(9) $${{µ}_{e}}_{1,2}=\frac{2{µ}_{g}cos\varphi \pm \sqrt{∆}}{4kT\frac{I_{u}{+I}_{v}}{I_{u}I_{v}}}$$

The molecular descriptors (dipole moments, polarizability, and ionization potential) in the ground electronic state of the solute can be estimated by quantum mechanical procedures [42,43].

The values of the correlation coefficients $C_{1}$ and $C_{2}$, determined in solvatochromic analyses, are not enough to solve Equations (3) and (4) with three unknown parameters (the excited state dipole moment and polarizability, and the angle φ between the dipole moments in the electronic states responsible for the absorption process).

McRae [3] supposes that the solute’s electric polarizability does not change its value in the absorption process. In this hypothesis, the system of the two equations can be solved with two variables: the excited state dipole moment and the angle φ. In order to obtain information about the excited state dipole moments of solute, the angle φ is varied until the excited state polarizability becomes equal to the ground state polarizability. The results obtained by the variation method [1,26,27] can be verified with the values given based on the model of pure liquid proposed by T. Abe [2].

The final equation of the Abe model shows that between the parameters *A* and *B* from (10) and (11), there exists a linear dependence, as expressed by relation (12).

(10) $$A=\frac{{µ}_{g}^{2}(v)+\frac{3}{2}\alpha_{g}(v)\frac{I_{g}(v)\left[I_{g}\left(u\right)-hc\nu_{s}\right]}{I_{g}\left(v\right)+I_{g}\left(u\right)-hc\nu_{s}}}{\frac{2}{3kT}{µ}_{g}^{2}\left(v\left(p\right)\right)+\alpha (v)}$$

(11) $$B=\frac{\frac{\nu_{0}-\nu_{s}}{C}+\left\{{µ}_{g}^{2}\left(v\right)+\frac{3}{2}\alpha_{g}(v)\frac{I_{g}\left(v\right)I_{g}\left(u\right)\left[I_{g}\left(u\right)\right]}{I_{g}\left(v\right)+I_{g}\left(u\right)}\right\}\;\alpha_{g}(u)}{\frac{2}{3kT}{µ}_{g}^{2}\left(v\left(p\right)\right)+\alpha (v)}$$

(12) $${µ}_{e}^{2}\left(u\right)-{µ}_{g}^{2}\left(u\right)+\alpha_{e}\left(u\right)A=B$$

In relation (11), the constant *C* can be computed as follows [2]:

$$C=\sum_{p}R_{uv(p)}^{-6}$$

(13) $$C=\frac{16\pi^{3}N_{A}^{2}}{9}{\left(\frac{\rho_{v}}{M_{v}}\right)}^{\frac{2}{3}}\left\{{\left[{\left(\frac{M_{u}}{\rho_{u}}\right)}^{\frac{1}{3}}+{\left(\frac{M_{v}}{\rho_{v}}\right)}^{\frac{1}{3}}\right]}^{-4}+{\left[{\left(\frac{M_{u}}{\rho_{u}}\right)}^{\frac{1}{3}}+{3\left(\frac{M_{v}}{\rho_{v}}\right)}^{\frac{1}{3}}\right]}^{-4}+{\left[{\left(\frac{M_{u}}{\rho_{u}}\right)}^{\frac{1}{3}}+5{\left(\frac{M_{v}}{\rho_{v}}\right)}^{\frac{1}{3}}\right]}^{-4}+\ldots \right\}$$

The following notations were made in relations (10)–(13): µ—electric dipole moment; α molecular polarizability; *I*—ionization potential; ν—wavenumber in the maximum of the electronic absorption band; *M*—molar mass; ρ—density; *T*—absolute temperature; *u* and *v* refer to the spectrally active molecule and to the solvent molecule, while *g* and *e* refer to the ground and excited electronic states, respectively; $N_{A}$ is Avogadro’s number; and *k* is the gaseous constant.

In dependence (12), *B* vs. *A*, the slope is the excited state polarizability of the solute molecule, and the intercept is the difference between the squares of the solute molecule dipole moments in the electronic states responsible for the light absorption process.

### 2.2. Spectral Analysis

**Figure 1:**
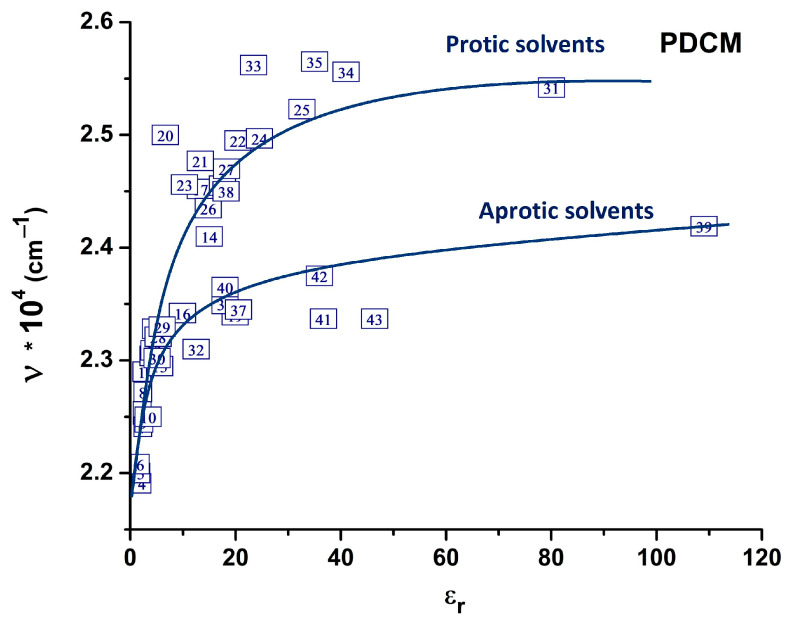
Wavenumber in the maximum of the ICT band of PDCM vs. the dielectric permittivity of the solvent (spectral data from Table 2).

**Figure 2:**
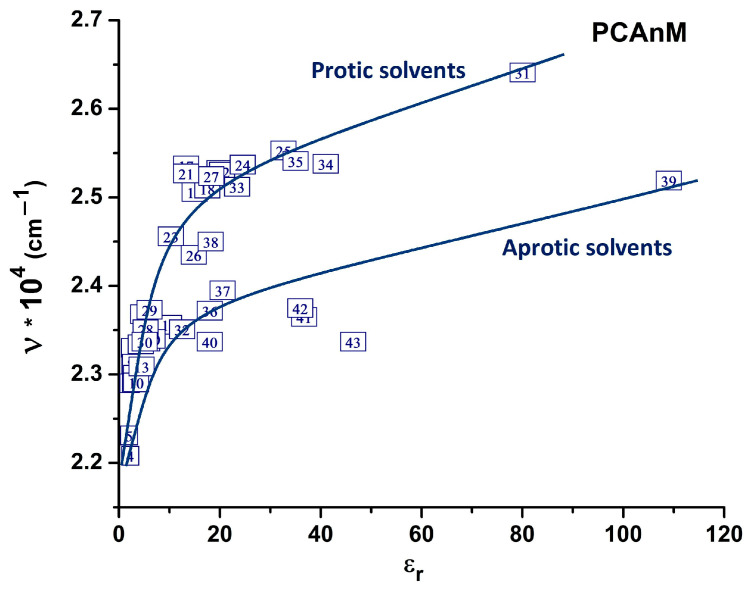
Wavenumber in the maximum of the ICT band of PCAnM vs. the dielectric permittivity of the solvent (spectral data from Table 3).

The analyzed molecules show intense electronic absorption UV bands due to *π* → π* transitions and a visible electronic band of low intensity that is very sensitive to the solvent characteristics, which is attributed to a n → π* transition [23,24]. The visible electronic absorption band of PDCM and PCAnM shifts to the blue in protic solvents, and when the polarity of the solvent increases due to the charge transfer from carbanion to the heterocycle. Table 1 lists the parameters used in the calculation of the excited state dipole by the variational method. The Abe parameters and the wavenumbers of the ICT band in the studied solvents are listed in Table 2 and Table 3. The blue shift of the ICT band of PDCM and PCAnM as a function of the dielectric permittivity of the solvent is represented in Figure 1 and Figure 2, respectively.

The graphs in Figure 1 and Figure 2 provide evidence of the action of specific interactions in protic solvents for which the wavenumbers in the maximum of the ICT band are shifted towards higher values compared with the values measured in aprotic solvents.

The statistical analysis of experimental data of PDCM and PCAnM using the solvent parameters was conducted, showing that the dispersive interactions described by the term $C_{2}f\left(n\right)$ and the specific interactions in which the solvent accepts protons were not significant [27,28]. The following equations describe the solvent’s influence on the ICT band of PDCM and PCAnM:

(14) $$\bar{v}\left({cm}^{-1}\right)=(21940\pm 130)+(1731\pm 210)f\left(Ɛ\right)+(1735\pm 120)\alpha$$

(for PDCM);

(15) $$\bar{v}\left({cm}^{-1}\right)=\left(22600\pm 143\right)+\left(1358\pm 401\right)f\left(Ɛ\right)+(1872\pm 203)\alpha$$

(for PCAnM).

The correlation coefficient C_1_ that multiplies the *f*(*ε*) parameter depends on the solute descriptors, as is shown in Equation (3).

The molecular descriptors for PDCM and PCAnM were determined by Spartan’14 software [42]. Equations (6) and (7), written using these molecular descriptors, are listed in the last column of Table 1. The real solutions for the excited state dipole moments of PDCM and PCAnM are also listed in the last column of Table 1. The intramolecular charge transfer of electrons from the carbanion towards the heterocycle takes place along the ylid bond $(\varphi =0^{{}^{\circ}}$). One can consider that the error in estimating the excited dipole moment of the studied molecules affects the first decimal.

**Table 1:** Parameters (computed with Spartan’14 and Density Functional EDF, 6-3131G*) used in the variational method for estimating the excited state dipole moment of the studied methylids.

Molecule	Parameter	Value	Equation and Results
PDCM	I_u_ (eV)	5.12	
	${µ}_{g}\;(D)$	3.94	$\alpha_{e}=61.5333-0.1290{µ}_{e}^{2}$
	$\alpha_{g}\;(A^{3})$	60.41	$0.0156{µ}_{e}^{2}-7.88{µ}_{e}cos\varphi \;$+ 24.09663 = 0
	${C_{1\;}(cm}^{-1})$	1371	${µ}_{e}=3.07$ D; $\varphi =0^{{}^{\circ}}$
	a (A)	2.7063	
PCAnM	I_u_ (eV)	5.24	
	${µ}_{g}\;(D)$	4.67	$\alpha_{e}=65.7770-0.1290{µ}_{e}^{2}$
	$\alpha_{g}\;(A^{3})$	63.71	$0.0156{µ}_{e}^{2}-9.34{µ}_{e}cos\varphi \;$+ 37.5862 = 0
	${C_{1}\;(cm}^{-1})$	1257	${µ}_{e}=4.05$ D; $\varphi =0^{{}^{\circ}}$
	a (A)	2.8507	

The dipole moment in the ground state of methylids was considered in toluene (I_v_ = 8.72 eV). From Table 1, the results show that, due to the absorption of a visible photon, the methylid molecules are excited in an electronic state with smaller dipole moment. Taking into account the approximation in which the spectral theory of solution was developed, the above results can be considered valid.

In the second part of this research, the model proposed by Takehiro Abe was considered for estimating the dipole moment in the first excited state of PDCM and PCAnM. By using Equations (10) and (11) and constant *C* determined according to Equation (13), the Abe parameters A and B were calculated, and their values are listed in Table 2 and Table 3. Also, the maxima of the ICT band of PDCM and PCAnM are included in the last column of Table 2 and Table 3.

**Table 2:** Abe parameters and wavenumbers for the maximum of ICT visible band of PDCM.

No.	Solvent	$10^{-44}C$	$10^{12}A$	$10^{36}B$	$\nu \;({cm}^{-1})$
1	Dioxane	1.94	4.42	379.33	22,900
2	p-Xylene	0.79	4.36	362.54	22,520
3	Benzene	1.19	4.46	381.32	22,550
4	CCl_4_	1.09	4.63	481.67	21,910
5	Cyclohexane	0.94	4.88	512.07	22,000
6	n-Heptane	0.62	4.65	464.53	22,080
7	Mesitylene	0.67	4.44	382.64	22,410
8	Toluene	0.95	3.68	283.33	22,720
9	o-Xylene	0.81	3.01	257.32	22,450
10	Trichloroethylene	1.95	1.90	187.63	22,500
11	Chloroform	1.34	127	76.52	23,280
12	Anisole	0.93	1.30	84.64	23,040
13	1,2 Dichloroetane	1.65	0.96	72.55	23,090
14	Cyclohexanol	0.99	1.09	13.67	24,100
15	Chlorobenzene	1.02	1.38	99.34	22,950
16	Dichloromethane	1.86	0.66	40.63	23,420
17	n-Hexyl alcohol	0.98	0.23	16.95	24,520
18	n-Butyl alcohol	1.17	0.73	−1.54	24,550
19	Methyl acetate	1.48	0.63	32.25	23,400
20	Iso-Propyl alcohol	1.48	0.58	−12.16	25,000
21	Benzyl alcohol	0.99	0.57	0.21	24,770
22	n-Propyl alcohol	1.52	0.56	−8.00	24,950
23	n-Octyl alcohol	0.75	1.10	16.85	24,560
24	Ethanol	2.09	0.52	−3.22	24,970
25	Methanol	3.30	0.31	−3.62	25,230
26	Pentanol	0.93	0.87	−12.58	24,350
27	Iso-Butyl alcohol	1.15	0.55	−1.41	24,700
28	Iso-Amyl acetate	0.62	1.05	53.31	23,210
29	Ethyl acetate	1.07	0.74	39.74	23,300
30	n-Butyl acetate	0.72	0.91	55.34	23,020
31	Water	4.11	0.17	3.55	25,420
32	Pyridine	1.37	0.19	−2.78	23,100
33	1,2 Propane diol	1.56	0.39	−4.82	25,620
34	1,2 Ethane diol	2.20	1.25	−2.75	25,560
35	1,3 Propane diol	1.59	0.28	−9.02	25,650
36	Methyl ethyl ketone	1.20	0.35	16.30	23,500
37	Acetone	2.02	0.26	15.22	23,450
38	Diacetone alcohol	0.72	0.33	−2.49	24,500
39	Formamide	3.37	0.13	6.51	24,190
40	DMF	1.33	0.19	8.37	23,650
41	Acetophenone	0.85	0.30	17.86	23,370
42	Acetonitrile	2.40	0.13	6.53	23,750
43	DMSO	1.62	0.18	9.24	23,370

The data in Table 2 were obtained using the following parameters of PDCM: $\nu_{0}=21,940{\;cm}^{-1};\;\rho =1.6149\;\frac{g}{{cm}^{3}}$; $I_{u}=5.12\;eV$; ${µ}_{g}=3.94\;D$; $\alpha_{g}=60.41\;A^{3};\;and\;M=237.26\;uam$. The value of *ν*_0_ (resulting from the statistical analysis) approximates the wavenumber in the maximum of the ICT band in vacuum. Because the methylids change their structure at high temperatures [23], we used the value of *ν*_0_ obtained by statistical means in Equation (14) for computing Abe parameters using the solvent data (in Table 4) and the maximum of the ICT band in Table 2.

**Table 3:** Abe parameters and wavenumbers in the maximum of ICT visible band of PCAnM.

No.	Solvent	$10^{-44}C$	$10^{12}A$	$10^{36}B$	$\nu \;({cm}^{-1})$
1	Dioxane	1.72	4.34	426.35	23,300
2	p-Xylene	0.71	4.28	390.38	23,110
3	Benzene	1.07	4.38	434.50	22,995
4	CCl_4_	0.98	4.63	481.67	22,080
5	Cyclohexane	0.84	4.88	512.07	22,310
6	n-Heptane	0.56	4.65	382.64	22,900
7	Mesitylene	0.86	4.44	382.64	22,900
8	Toluene	0.86	3.25	345.14	23,120
9	o-Xylene	0.73	3.25	330.74	22,995
10	Trichloroethylene	1.09	4.29	505.22	22,910
11	Chloroform	1.19	1.25	80.08	23,680
12	Anisole	0.84	1.28	103.14	23,340
13	1,2 Dichloroetane	1.46	0.96	95.18	23,090
14	Cyclohexanol	0.89	1.07	17.23	25,060
15	Chlorobenzene	0.91	1.16	99.28	23,370
16	Dichloromethane	1.63	0.66	53.41	23,560
17	n-Hexyl alcohol	0.69	0.93	−28.76	25,360
18	n-Butyl alcohol	1.05	0.65	0.76	25,100
19	Iso-Propyl alcohol	1.32	0.56	−8.72	25,310
20	Methyl acetate	1.33	0.63	50.30	23,400
21	Benzyl alcohol	0.89	0.84	−0.24	25,270
22	n-Propyl alcohol	1.36	0.55	−4.52	25,290
23	n-Octyl alcohol	0.51	1.10	13.69	24,560
24	Ethanol	1.86	0.44	−1.87	25,370
25	Methanol	2.91	0.31	−1.96	25,530
26	Pentanol	0.84	1.03	42.36	24,350
27	iso-Butyl alcohol	1.03	0.65	−2.68	25,240
28	Iso-Amyl acetate	0.55	0.96	63.44	23,510
29	Ethyl acetate	1.69	0.73	63.43	23,730
30	n-Butyl acetate	0.65	0.90	69.68	23,370
31	Water	0.76	0.18	−16.42	26,410
32	Pyridine	1.23	0.18	0.70	23,510
33	1,2 Propane diol	1.39	0.40	9.47	25,120
34	1,2 Ethane diol	1.86	0.30	2.75	25,380
35	1,3 Propane diol	1.43	0.28	−0.90	25,410
36	Methyl ethyl ketone	1.04	0.32	19.23	23,720
37	Acetone	0.82	0.25	3.40	23,950
38	Diacetone alcohol	0.71	0.33	9.14	24,500
39	Formamide	2.99	0.13	5.54	25,190
40	Acetophenone	0.77	0.30	25.00	23,370
41	DMF	1.28	0.19	12.99	23,650
42	Acetonitrile	21.36	0.13	12.99	23,750
43	DMSO	1.45	0.18	11.61	23,370

The data in Table 3 were obtained with the following parameters of PCAnM [14]: $\nu_{0}=22,600\;{cm}^{-1}$, $\rho =1.561\frac{g}{{cm}^{3}}$; $I_{u}=5.24\;eV$; ${µ}_{g}=4.67\;D$; and $\alpha_{g}=63.71\;A^{3};\;M=268.165\;uam$. The value of *ν*_0_ results from statistical analysis and approximates the maximum of the ICT band in vacuum. Similarly, with the PDCM case, the *ν*_0_ value obtained by statistical means in Equation (15) was considered as the value of the ICT wavenumber for the isolated molecule. The Abe parameters were computed with the solvent data from Table 5 and the maxima of the ICT band from Table 3.

**Table 4:** The solvent parameters for the Abe model.

No.	Solvent	${µ}_{g}\;(D)$	$\alpha_{g}\;({Å}^{3})$	$I_{g}\;(eV)$	M (g/mol)	$\rho \;(\frac{g}{{cm}^{3}})$
1	Dioxane	0.00	9.44	9.52	88.11	1.417
2	p-Xylene	0.00	14.35	8.52	106.17	0.862
3	Benzene	0.00	10.44	9.25	78.11	0.868
4	CCl_4_	0.00	10.5	9.72	153.82	1.594
5	Cyclohexane	0.00	10.85	11.0	84.16	0.779
6	n-Heptane	0.00	13.61	10.35	100.2	0.683
7	Mesitylene	0.00	16.12	8.76	120.20	0.864
8	Toluene	0.38	12.4	8.72	92.14	0.867
9	o-Xylene	0.64	14.25	8.56	106.17	0.880
10	Trichloroethylene	0.80	9.75	9.45	131.4	1.460
11	Chloroform	1.15	8.23	11.50	119.38	1.446
12	Anisole	1.38	13.10	8.20	108.14	0.995
13	1,2 Dichloroethane	1.43	8.68	10.49	173.84	2.447
14	Cyclohexanol	1.46	11.94	10.0	100.16	0.962
15	Chlorobenzene	1.50	13.0	9.07	112.56	1.110
16	Dichloromethane	1.60	6.66	11.32	84.93	1.330
17	n-Hexyl alcohol	1.60	12.4	8.98	102.175	0.814
18	n-Butyl alcohol	1.66	8.88	9.99	74.12	0.810
19	Iso-Propyl alcohol	1.66	6.67	9.90	60.1	0.786
20	Methyl acetate	1.67	6.99	10.51	74.08	0.972
21	Benzyl alcohol	1.67	11.89	8.26	108.14	1.044
22	n-Propyl alcohol	1.68	6.67	10.52	60.10	0.803
23	n-Octyl alcohol	1.68	16.1	9.8	130.227	0.827
24	Ethanol	1.69	5.06	10.70	46.07	0.789
25	Methanol	1.70	3.21	10.85	32.04	0.792
26	Pentanol	1.70	11.58	10.42	88.15	0.814
27	Iso-Butyl alcohol	1.76	9.07	10.12	74.12	0.802
28	Iso-Amyl acetate	1.77	15.18	9.90	130.18	0.884
29	Ethyl acetate	1.78	9.70	10.11	88.11	0.902
30	n-Butyl acetate	1.84	13.42	10.00	116.16	0.883
31	Water	1.85	1.50	12.59	18	1.000
32	Pyridine	2.20	2.41	9.34	79.1	0.978
33	1,2 Propane diol	2.27	8.01	10.00	76.10	1.036
34	1,2 Ethane diol	2.28	5.48	10.55	62.07	1.11
35	1,3 Propane diol	2.53	6.50	10.42	76.10	1.060
36	Methyl ethyl ketone	2.76	8.28	9.54	72.11	0.805
37	Acetone	2.80	6.27	9.89	58.08	0.971
38	Diacetone alcohol	3.24	12.4	9.6	116.16	0.938
39	Formamide	3.73	4.08	10.20	45.04	1.133
40	Acetophenone	3.81	14.37	9.77	120.14	1.028
41	DMF	3.86	7.91	9.12	73.94	0.944
42	Acetonitrile	3.92	4.30	12.20	41.05	0.786
43	DMSO	4.1	8.0	9.10	78.13	1.100

**Table 5:** The solvent parameters.

No.	Solvent	ε_r_	n	α	β	π*
1	Dioxane	2.21	1.4224	0.00	0.37	0.49
2	p-Xylene	2.28	1.4958	0.00	0.12	0.43
3	Benzene	2.27	1.5011	0.00	0.10	0.59
4	Carbon tetrachloride	2.24	1.4601	0.00	0.10	0.28
5	Cyclohexane	2.02	1.4266	0.00	0.00	0.00
6	n-Heptane	1.92	1.3855	0.00	0.00	−0.08
7	Mesitylene	2.40	1.499	0.00	0.13	0.41
8	Toluene	2.38	1.4969	0.00	0.11	0.49
9	o-Xylene	2.60	1.5054	0.00	0.16	0.48
10	Trichloroethylene	3.40	1.4767	0.00	0.05	0.48
11	Chloroform	4.81	1.4459	0.20	0.10	0.53
12	Anisole	4.33	1.517	0.00	0.32	0.73
13	1,2 Dichloroethane	4.50	1.5389	0.10	0.10	0.48
14	Cyclohexanol	13.4	1.465	0.66	0.84	0.45
15	Chlorobenzene	5.60	1.5241	0.00	0.07	0.71
16	Dichloromethane	9.93	1.4242	0.13	0.10	0.82
17	n-Hexyl alcohol	13.3	1.418	0.55	0.32	0.13
18	n-Butyl alcohol	17.51	1.393	0.84	0.84	0.47
19	Methyl acetate	6.68	1.3614	0.00	0.42	0.60
20	Iso-Propyl alcohol	19.92	1.3772	0.76	0.84	0.48
21	Benzyl alcohol	13.3	1.5396	0.60	0.52	0.98
22	n-Propyl alcohol	20.45	1.3856	0.84	0.90	0.52
23	n-Octyl alcohol	10.3	1.429	0.54	0.32	0.14
24	Ethanol	24.55	1.3614	0.86	0.75	0.54
25	Methanol	32.63	1.3314	0.98	0.66	0.6
26	Pentanol	14.8	1.409	0.54	0.49	0.15
27	Iso-Butyl alcohol	18.3	1.3943	0.54	0.31	0.15
28	Iso-Amyl acetate	5.3	1.398	0.00	0.45	0.46
29	Ethyl acetate	6.08	1.3723	0.00	0.45	0.55
30	n-Butyl acetate	5.1	1.395	0.00	0.45	0.46
31	Water	80.04	1.33	1.20	0.50	1.20
32	Pyridine	12.5	1.5093	0.00	0.7	0.9
33	1,2 Propane diol	23.4	1.4324	0.83	0.78	0.76
34	1,2 Ethane diol	41	1.432	0.90	0.52	0.92
35	1,3 Propane diol	35	1.4398	0.80	0.77	0.84
36	Methyl ethyl ketone	18	1.3793	0.06	0.48	0.60
37	Acetone	20.56	1.3855	0.08	0.48	0.62
38	Diacetone alcohol	18.2	1.4232	0.00	0.45	0.72
39	Formamide	109	1.4475	0.71	0.48	0.97
40	N,N-Dimethylformamide	18	1.4305	0.00	0.76	0.88
41	Acetophenone	36.71	1.534	0.04	0.49	0.81
42	Acetonitrile	35.94	1.3441	0.19	0.40	0.66
43	Dimethyl sulfoxide	46.45	1.4793	0.00	0.76	1.00

**Figure 3:**
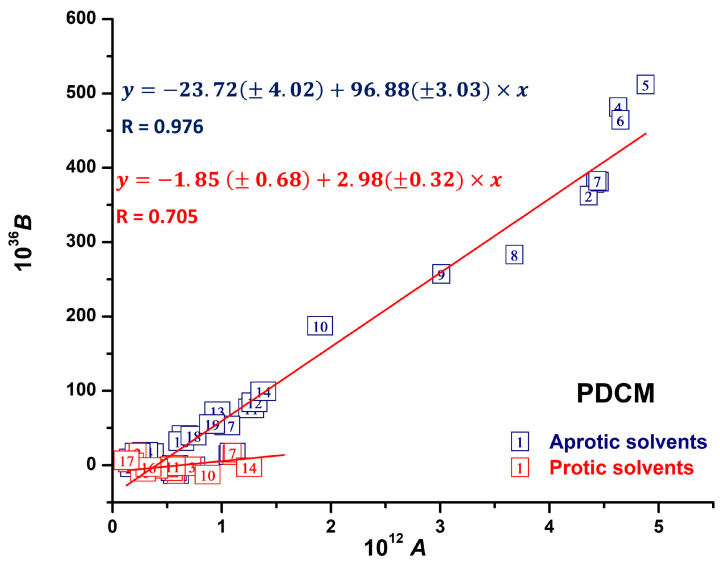
The graphical representation of the B vs. A dependency for PDCM in all solvents using the data from Table 2.

**Figure 4:**
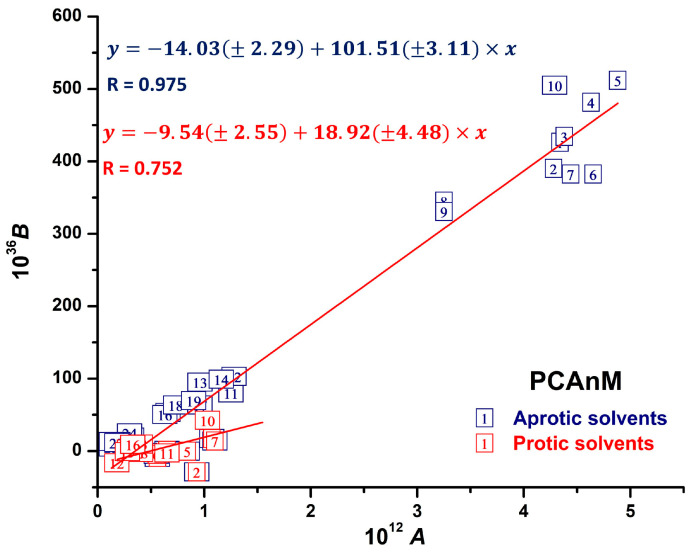
The graphical representation of the B vs. A dependency for PCAnM in all solvents using the data from Table 3.

The dependencies B vs. A of the Abe parameters for PDCM and PCAnM are plotted in Figure 3 and Figure 4 for all solvents from Table 2 and Table 3, respectively. One can see that the points corresponding to the protic solvents are located at the beginning of the line and are clearly separated from the rest of the solvents.

As observed in Figure 3, for PDCM, the linear fit of experimental data gives Equation (16) for aprotic solvents:

(16) $$B=-23.72\left(\pm 4.02\right)+96.88\left(\pm 3.03\right)A$$

From (16), the following is obtained: ${µ}_{e}^{2}-{µ}_{g}^{2}=-23.72$. The dipole moment of PDCM in the ground state in toluene is computed as ${µ}_{g}=3.94\;D$; therefore, ${µ}_{e}^{2}=-8.196\;D$. This value is unacceptable from a mathematical point of view. By using the ground state dipole moment of PDCM calculated by Spartan’14 in water, ${µ}_{g}=6.28\;D$, and it results in ${µ}_{e}=3.96\;D$. Therefore, the Abe model applied to PDCM in aprotic solvents gives a lower value for the dipole moment in the excited state than the value calculated for the ground state in water.

For PDCM in protic solvents, the linear fit of experimental data in Figure 3 gives Equation (17):

(17) $$B=-1.85\left(\pm 4.02\right)+2.98\left(\pm 3.03\right)A$$

In this case, the value of μ*_g_* calculated for water (6.28 D) must be used for determining the dipole moment in the excited state, μ*_e_*, where the condition ${µ}_{e}^{2}-{µ}_{g}^{2}=-1.85\;D$ is valid. It results ${µ}_{e}=6.13\;D$, a value that is lower than the dipole moment in the ground state, which is in accordance with the experimental data. The small variation in the dipole moment of PDCM in protic solvents during absorption could be explained by the intermolecular hydrogen bond formation with the solvent.

An analysis of the plots in Figure 4 for PCAnM gives the linear fitting for aprotic solvents as follows:

(18) $$B=-14.03\left(\pm 4.02\right)+98.88\left(\pm 3.03\right)A$$

For ${µ}_{e}^{2}-{µ}_{g}^{2}=-14.03$ and by using the value ${µ}_{g}=4.67\;D$ (the dipole moment of PCAnM in the ground state calculated in toluene), the ${µ}_{e}=2.79\;D$ value is determined. Here, the Abe model applied for aprotic solvents gives a dipole moment in the excited state that is lower than that in the ground state. This result is in agreement with the intramolecular charge transfer that occurs during absorption.

For PCAnM in protic solvents, the linear fit of experimental points is described using Equation (19):

(19) $$B=-9.54\left(\pm 2.55\right)+18.92\left(\pm 4.48\right)A$$

Using the calculated value of the ground state dipole moment in water by Spartan’14, which is ${µ}_{g}=5.61\;D$, the dipole moment in the excited state of PCAnM is obtained as ${µ}_{e}=4.68\;D$.

By analyzing the above results for both PDCM and PCAnM, the Abe model shows that the polarizability does not remain unchanged in the absorption process. Instead, a large difference is observed in polarizability between the ground and the excited state.

Although in the Abe model, the specific interactions are neglected, and the electric dipole moments in the transition states are considered as collinear, the results obtained on its base indicate a difference between the electric polarizabilities in transition electronic states. Therefore, the variational method could be only estimative in the absence of a third possibility for estimating the dipole moment in the excited state of molecules with only absorption spectra.

Figure 3 and Figure 4 also suggest that the Abe model does not correctly describe the influence of the protic solvents on the wavenumbers in the maximum of the visible absorption band of zwitterionic molecules, such as methylids.

The results of the statistical analysis of the experimental data based on Equation (2) were used in order to eliminate the influence of the specific interactions on the wavenumber of the visible ICT band of PDCM and PCAnM. Relations (14) and (15), obtained in statistical analysis, allowed the contribution of these interactions to the spectral shifts in hydroxy solvents to be estimated.

Accordingly, the correlation coefficients calculated for PDCM and PCAnM are given in Equations (14) and (15). Using the values of *C*_4_ and the empirical coefficient *α*, the contribution of the hydrogen bond between the protic solvents and the methylids, ${∆\nu }_{sp.}$ was determined. The values of ${∆\nu }_{sp}$ are included in the last column in Table 6 and Table 7.

Next, the $\nu_{univ}$ parameter was introduced as being the difference between the measured wavenumber of the maximum in the ICT band and the spectral shift $\nu_{sp}$ due to the specific interactions of intermolecular hydrogen bonding. The $\nu_{univ}$ values contain only the contribution of the universal interactions to the ICT band wavenumber. The maximum of the ICT band that does not contain the spectral shift arising from specific interactions, i.e., $\nu_{univ}$, is listed in the last column of Table 6 and Table 7.

**Table 6:** Abe parameters for PDCM in hydroxy solvents.

No.	Solvent	α	$\nu_{sp.}$	$10^{-44}C$	$10^{12}A$	$10^{36}B$	$\nu_{univ.}\;({cm}^{-1})$
14	Cyclohexanol	0.66	1145	0.99	1.17	59.91	23,224
17	n-Hexyl alcohol	0.80	1388	0.77	0.98	53.96	23,132
18	n-Butyl alcohol	0.84	1457	1.17	0.77	41.00	23,093
19	Iso-Propyl alcohol	0.76	1320	1.48	0.61	21.28	23,680
21	Benzyl alcohol	0.60	1041	0.99	0.90	35.34	23,730
22	n-Propyl alcohol	0.84	1457	1.52	0.60	27.77	23,493
23	n-Octyl alcohol	0.77	1336	0.57	0.52	71.42	23,224
24	Ethanol	0.86	1492	2.09	0.48	27.33	23,478
25	Methanol	0.98	1700	3.30	0.49	16.50	23,530
26	Pentanol	0.84	1457	0.94	30.47	59.22	23,893
27	Iso-Butyl alcohol	0.69	1197	1.16	0.71	36.04	23,503
31	Water	1.17	2030	4.11	0.18	3.64	23,570
33	1,2-Propane diol	1.10	1908	1.56	0.50	13.61	23,410
34	1,2-Ethane diol	0.90	1562	2.20	0.31	12.70	23,998
35	1,3-Propane diol	1.21	2100	1.59	0.31	14.55	23,520
38	Diacetone alcohol	0.65	1128	0.78	0.35	12.83	23,372
39	Formamide	0.71	1320	3.37	0.14	9.68	22,960

**Table 7:** Abe parameters for PCAnM in hydroxy solvents.

No.	Solvent	α	${∆\nu }_{sp.}$	$10^{-44}C$	$10^{12}A$	$10^{36}B$	$\nu_{univ.}\;({cm}^{-1})$
14	Cyclohexanol	0.66	1229	0.88	1.13	75.77	23,831
17	n-Hexyl alcohol	0.80	1490	1.17	1.00	77.04	23,870
18	n-Butyl alcohol	0.84	1564	1.05	0.71	55.38	23,536
19	Iso-Propyl alcohol	0.76	1415	1.33	0.59	32.12	23,895
21	n-Benzyl alcohol	0.60	1117	0.89	0.88	42.75	24,153
22	n-Propyl alcohol	0.84	1564	1.36	0.60	38.69	23,726
23	n-Octyl alcohol	0.77	1434	0.51	1.49	130.27	23,126
24	Ethanol	0.86	1600	1.86	0.48	30.95	23,770
25	Methanol	0.98	1825	2.92	0.33	22.48	23,705
26	Pentanol	0.84	1564	0.84	0.94	98.49	22,786
27	Iso-Butyl alcohol	0.69	1285	1.03	0.69	37.27	23,955
31	Water	1.17	2179	7.63	0.17	10.87	24,231
33	1,2-Propane diol	1.10	2048	1.38	0.44	40.96	23,072
34	1,2-Ethane diol	0.90	1676	1.96	0.32	23.16	23,704
35	1,3-Propane diol	1.21	2253	1.42	0.32	27.17	23,157
38	Diacetone alcohol	0.65	1210	0.71	0.35	27.31	23,290
39	Formamide	0.71	1322	2.99	0.13	9.31	23,868

**Figure 5:**
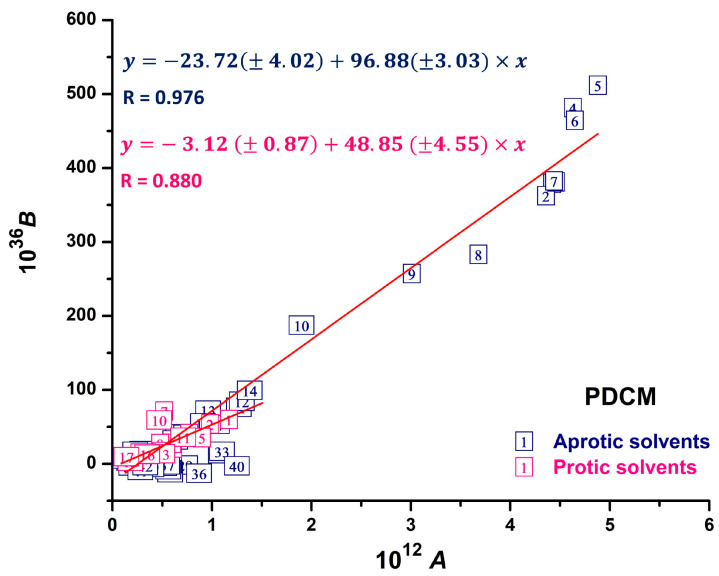
Dependence of *B* as a function of *A* for PDCM in all solvents when the contribution of intermolecular hydrogen bonding to the spectral shift was eliminated.

**Figure 6:**
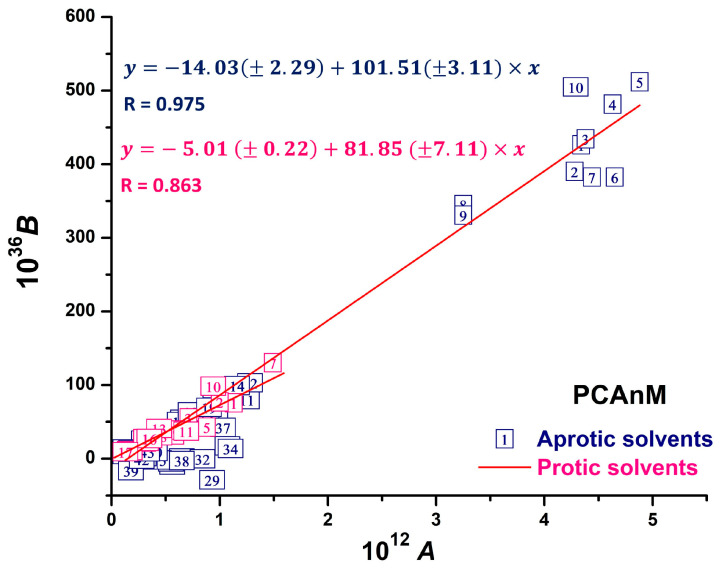
Dependence of *B* as a function of *A* for PCAnM in all solvents when the contribution of intermolecular hydrogen bonding to the spectral shift was eliminated.

Using the obtained values $\nu_{univ.}$ for the maximum of the ICT band of PDCM and PCAnM that does not contain the contribution of specific interactions, ${∆\nu }_{sp},$ the Abe parameters were recalculated. Therefore, the contribution of specific interactions was eliminated from the values of the new *A* and *B* parameters. With these values, plotted in Figure 5 and Figure 6 for PDCM and PCAnM, respectively, the dipole moment in the excited state and the polarizability were estimated for both methylids.

The graphs in Figure 5 and Figure 6 demonstrate the applicability of the Abe model to solutions where the specific interactions have a low or no contribution at all.

A very good linear dependence between the Abe parameters *B* and *A* was obtained in these conditions for both PDCM and PCAnM. Moreover, by using the Abe model to estimate the dipole moment in the excited state, values were obtained that were lower than those in the ground state. These values are in agreement with the variational model and with the experimental hipsochromic shift of the ICT band in protic and polar solvents compared to the non-polar solvents.

The Abe model provides evidence of the variations in polarizability during absorption of visible light. For both PDCM and PCAnM, the estimated values of the excited state polarizability (in the limits of this model) are higher than those in the ground state. In the case of protic solvents, the methylids that are intermolecularly H-bonded with the solvent molecules have a polarizability in the excited state smaller than that in the ground state.

### 2.3. The Ability of PDCM and PCAnM to Discriminate the Solvents

Based on the solvatochromic response of the ICT band, we further analyzed the ability of PDCM and PCAnM to discriminate the solvents, using the principal component analysis (PCA) as a statistical method [44,45,46,47]. The solvatochromic response matrix was constructed from the wavenumber maximum of the ICT band, $\tilde{\nu }$, and the f(*ε*) *α*, *β* and π* parameters of the solvent extracted from the above-reported study. In the first stage, all forty-three solvents were used as the learning matrix.

**Figure 7:**
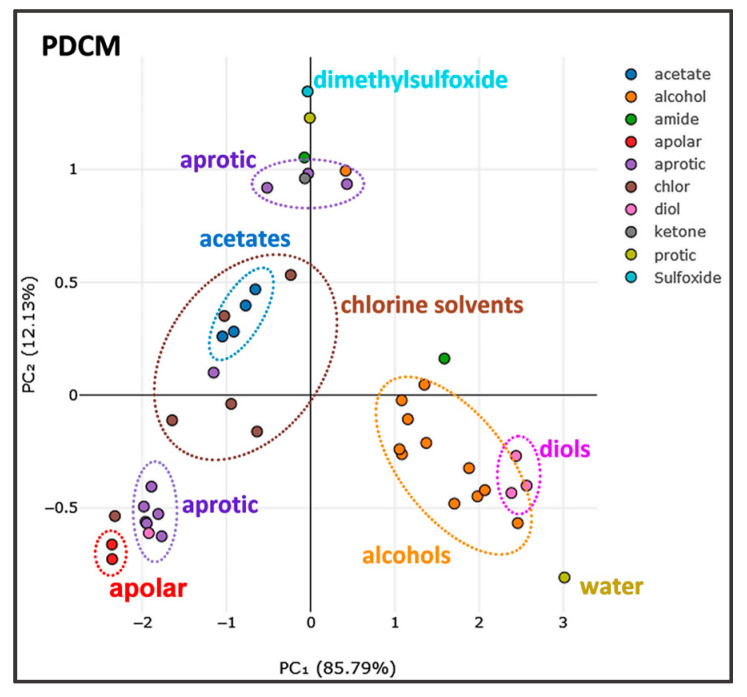

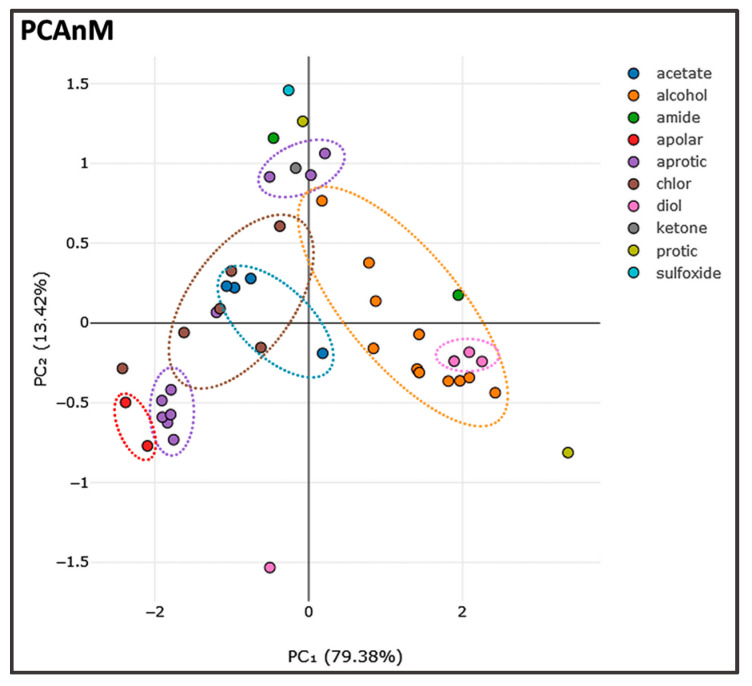
The two-dimensional PCA score plots of PDCM (**top**) and PCAnM (**down**) for the discrimination of the forty-three solvents used in the solvatochromic analysis. The sensing parameters are the maximum of the ICT band, $\tilde{\nu }$, and the solvent parameters are *f*(*ε*) and *α*. The color code and solvent assignments for PCAnM are identical to those given for PDCM.

When the whole set of the sensing parameters was subjected to PCA, the scree plot showed that the first two principal components out of five covered approx. 85% of the total variance of the data for both PDCM and PCAnM. The first principal component accounted for about 66%, while the second component carried around 18% of the variance data. By repeatedly narrowing the set of the solvent parameters, we obtained the best segregation when only $\tilde{\nu }$, f(*ε*) and *α* were subjected to PCA. As illustrated in Figure 7, the first two components had eigenvalues of more than 0.95 for both PDCM and PCAnM. The first component was dominant and covered more than 85% of the total variance of the data for PDCM and 79% for PCAnM; the second one accounted for ≈12%, and the third one represented 2% and 7%, respectively.

The two-dimensional plots in Figure 7 for the first two principal components provide several well-separated clusters for alcohols, acetates, chlorine solvents, or diols. In each area, the corresponding solvents generate distinct solvatochromic patterns. Water stood as an outlier irrespective of which set of parameters was used. Methyl ethyl ketone, pyridine or acetophenone formed a distinct cluster, as they produced similar responses to the ICT band. Similar PCA results were obtained when *α* was replaced with *β*. In this context, we concluded that, for these kinds of zwitterionic molecules, the most sensitive elements for identification and discrimination of the solvent type from a large set of data that work with the wavenumber of the ICT band are the hydrogen donating and accepting abilities of the solvents.

Next, we tested the performance of PDCM and PCAnM as solvatochromic sensors for identifying binary solvent mixtures from the rest of the studied solvents. The ethylene–glycol–dioxane mixture (EG + dioxane) was chosen for PDCM, where the solvent composition, the dielectric permittivity, and the maximum of the ICT band for every volume ratio were taken from a previous study [48]. We extended the list of the need parameters by calculating f(*ε*), *α*, *β*, and π* of each ratio in the solvent mixture. The final set of parameters for binary solvents prepared for PCA discriminatory investigation is reported in Table 8.

The water and ethanol (W + EtOH) and water and methanol (W + MeOH) alcohol mixtures were chosen from a previously reported study for PCAnM, with different volume ratios [28]. Table 9 gives the composition and the solvent parameters for PCAnM in the two alcohol mixtures.

**Table 8:** The ethylene glycol volume fraction, x_EG_, the parameters of the ethylene glycol–dioxane mixtures, and the maximum of the ICT band of PDCM.

No. crt	x_EG_^a^	A ^b^	Β ^b^	π* ^b^	*f*(ε) ^b^	${\tilde{\nu }}_{PDCM}$ ^a^
1	0	0	0.37	0.55	0.28571	22,640
2	0.05	0.045	0.3775	0.5685	0.44444	22,700
3	0.1	0.09	0.385	0.587	0.57143	22,810
4	0.2	0.18	0.4	0.624	0.72727	23,090
5	0.3	0.27	0.415	0.661	0.80132	23,360
6	0.4	0.36	0.43	0.698	0.83696	24,040
7	0.5	0.45	0.445	0.735	0.86547	24,305
8	0.6	0.54	0.46	0.772	0.8855	24,570
9	0.7	0.63	0.475	0.809	0.9	24,760
10	0.8	0.72	0.49	0.846	0.91202	24,980
11	0.9	0.81	0.505	0.883	0.92063	25,210
12	1	0.9	0.52	0.92	0.93023	25,560

^a^ value taken from Ref. [48]; ^b^ values calculated in this work.

**Table 9:** The water volume x_water_ in the water + ethanol, and water + methanol binary solvents, the corresponding solvents parameters, and the maximum of the ICT band of PCAnM [28].

No. crt.	Binary Solvent	x_water_	α	β	π*	*f*(ε)	${\tilde{\nu }}_{PCAnM}$
1	water + ethanol	0	0.83	0.98	0.51	0.221	25,370
2		0.1	0.84	0.96	0.57	0.888	25,460
3		0.2	0.83	0.93	0.63	0.899	25,570
4		0.3	0.82	0.92	0.68	0.904	25,682
5		0.4	0.8	0.91	0.73	0.912	25,760
6		0.5	0.79	0.9	0.77	0.921	25,840
7		0.6	0.77	0.89	0.82	0.93	25,880
8		0.7	0.74	0.88	0.9	0.939	25,920
9		0.8	0.67	0.87	1	0.948	25,946
10		0.9	0.59	0.97	1.11	0.956	25,965
11		1	0.5	1.26	1.13	0.204	25,980
12	water + methanol	0.1	0.74	1.12	0.64	0.923	25,608
13		0.2	0.74	1.09	0.7	0.931	25,685
14		0.3	0.74	1.06	0.76	0.938	25,750
15		0.4	0.72	1.04	0.82	0.943	25,810
16		0.5	0.7	1.03	0.88	0.948	25,854
17		0.6	0.66	1.01	0.95	0.951	25,890
18		0.7	0.63	1.01	1.01	0.954	25,918
19		0.8	0.59	1.06	1.06	0.958	25,942
20		0.9	0.54	1.09	1.11	0.961	25,965
21		1	0.49	1.23	1.14	0.204	25,980

**Figure 8:**
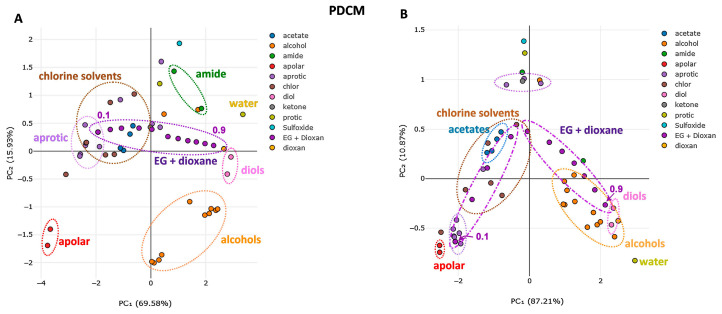
Discrimination of different volume ratios of the binary mixture ethylene glycol + dioxan (EG + dioxan) using PDCAM and two different sets of solvatochromic sensing elements: (**A**)—the maximum of the ICT band, $\tilde{\nu }$, *f*(*ε*), and the *α*, *β* and π* parameters of the solvent; (**B**)—with $\tilde{\nu }$, *f*(*ε*), and *α*.

**Figure 9:**
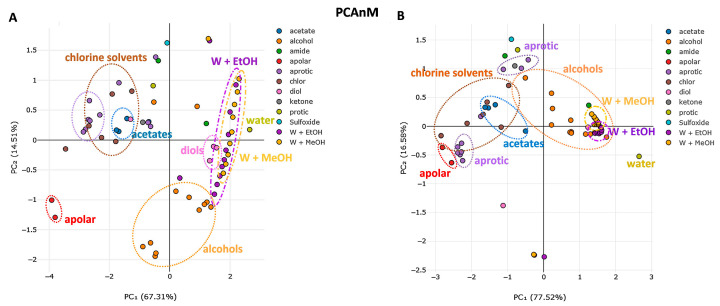
Discrimination of different volume ratios of the binary mixtures water + ethanol (W + EtOH) and water + methanol (W + MeOH) using PCAnM and two different sets of solvatochromic sensing elements: (**A**)—the maximum of the ICT band, $\tilde{\nu }$, *f*(*ε*), and the *α*, *β* and π* parameters of the solvent; (**B**)—with $\tilde{\nu }$, *f*(*ε*), and *α*.

The response pattern of PDCM and PCAnM with binary mixtures reported in Table 8 and Table 9, along with the whole set of forty-three solvents, are visualized in the PCA plots in Figure 8 and Figure 9. The first two principal components of PDCM and PCAnM were 85% and 82%, respectively, from the total variance when the whole set of sensing elements was used in the analysis (Figure 8A and Figure 9A). The pattern of the EG + dioxane mixture in PDCM in Figure 8A spreads along the two quadrants of the plot. A similar distribution was obtained for the W + EtOH and W + MeOH points for PCAnM (Figure 9A). At first sight, the location of these clusters seemed to be determined by an equilibrium between the protonation of the ylide and the proton-donating ability of the solvent, and this phenomenon was clearly observed in the binary mixtures.

Still, the sensitivity of either PDCM or PCAnM to the ratio between the two solvents in the binary mixture decreased when the π* polarizability parameter was removed from the analysis (Figure 8B and Figure 9B). This trend is even better highlighted for PCAnM (Figure 9B), where the points in the W + EtOH and W + MeOH clusters cannot be individually identified due to the high overlap. So, the discrimination power is less affected when the solvent polarizability is not included in the analysis of a polar protic—aprotic solvent mixture. When a mixture of two strong protic solvents is investigated, the discrimination power of the solute is lost if the polarizability of the solvent is removed. This points to the fact that, for zwitterionic molecules, the solvatochromic response, especially to strong interacting solvents, is a complex function of intermolecular proton transfer processes and polarization phenomena.

When a sufficiently large set of sensing elements is used, the two methylids, PDCM and PCAnM, are able to distinguish different types of mixtures with a complex composition and at any volume ratio with high accuracy. This sensitivity might be applied to the detection of harmful reagents in contaminated waters or in organic solvents.

## 3. Materials and Methods

Two carbanion disubstituted pyridinium methylids were chosen to verify the applicability of the spectral procedures to estimate the excited dipole moments of the solute molecules.

**Scheme 1:**
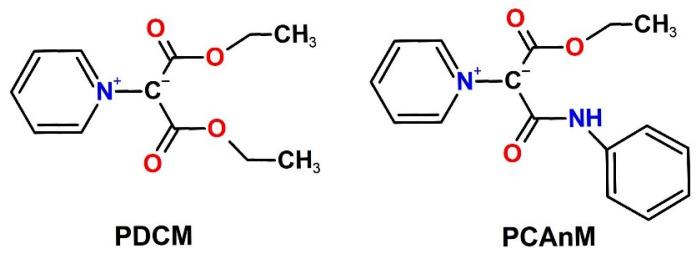
Structural formulae of the studied methylids: (PDCM—pyridinium dicarbethoxy methylid; PCAnM—pyridinium carbethoxy anilido methylid).

The salt method [23] was used for their preparation. Both pyridinium methylid’s structural features and purity were checked by spectral (^1^H-NMR and IR) and chemical methods. The structural formulae of the studied methylids are given in Scheme 1.

Some information about the molecular descriptors and also some values for wavenumbers in the maximum of the ICT visible band in a part of solvents are available from the literature [27,28] for the chosen carbanion and disubstituted pyridinium methylids for PDCM and PCAnM. For this study, the number of solvents in which the visible spectrum is recorded was increased using the liquids that solve the solutes, and which have known parameters asked for applying the Abe model.

The electronic absorption spectra were recorded with a Specord UV Vis spectrophotometer Carl Zeiss Jena with a data acquisition system. The wavenumbers in the maximum of the visible absorption band with ICT were measured with a precision of about $\pm 5\;{cm}^{-1}$ in 43 solvents with spectral purity.

The data for the molecular descriptors of the studied molecules are given in [27,28]. The solvent parameters used in our research are listed in Table 4 and Table 5.

The solvent parameters listed in Table 4 and Table 5 were used when computing the Abe parameters, *A* and *B*, for the studied methylids and to make a statistical analysis based on relation (2). As it is shown in ref. [41], the π* parameter linearly depends on the solvent functions *f*(ε) and *f*(*n*) and was neglected in this study.

## 4. Conclusions

The aim of this study is to decide if the two described models can be applied in estimating the excited state dipole moment of molecules that are spectrally active only in absorption and lack fluorescence. Both models used to estimate the dipole moment in an excited state were developed from simplified hypotheses, and the obtained results can be considered as being only informative.

In spite of the different hypotheses introduced in both models, in order to avoid the complexity of the liquid state, a common conclusion can be drawn, namely, the fact that the dipole moment in the excited state of both methylids decreases in the visible photon absorption. These results are in agreement with the transition of the electronic charge from the carbanion to the heterocycle.

Important differences between the two methods used to estimate the excited dipole moment of the studied zwitterionic molecules appear when characterizing the polarizability of the excited state. The restrictive hypothesis that the electric polarizability of one molecule does not change (or changes in neglectable quantities) in the visible photon absorption was confirmed by the results obtained with the Abe procedure, which indicates the increase in electric polarizability by excitation for the case of solutions achieved in non-protic solvents.

In aprotic solvents, the polarizability of methylids in the excited state computed by the Abe model is higher than in the ground state, while the variational model supposes that the polarizability remains unchanged during absorption in the visible domain.

When the supplementary contribution of the specific interactions is eliminated, the Abe model gives very good linear dependence between the calculated parameters A and B and also provides the values for both the dipole moment and the polarizability in an excited state.

In order to decide what method is applicable to estimate the excited state dipole moments of the non-fluorescent molecules, more studies must be made both with different solute molecules and a greater number of solvents with different physical-chemical parameters.

Finally, the sensitive solvatochromic responses of PDCM and PCAnM were tested for discriminating solvents and binary solvent mixtures. The principal component analysis demonstrates the ability of the studied methylids to participate in proton changes in protic solvents and also to distinguish between different types of mixtures (with a complex composition and at any volume ratio) with high accuracy.

## Data Availability

The data presented in this study will be available on request from the corresponding author.
